# Pumpkin Seed in Tenia

**Published:** 1855-01

**Authors:** 


### TREATMENT OF TENIA BY THE SEEDS OF CUCURBITA PEPO (PUMPKIN SEED.)

The seed of the pumpkin, a highly valued remedy of tapeworm, and one that has been the most fully tested, though not discovered by Americans, seems to be altogether ignored by American compilers of Dispensatories and Formularies.

The pumpkin seed remedy is often mentioned as being a new one, and as having been first introduced into practice in America. It appears, however, that this article was first introduced to the notice of the medical world by Dr. Mongeney, of Bordeaux, about three years ago, at a meeting of the Medical Society of that city; he then and there declared that for thirty years he had used with great success for the expulsion of tænia, a paste of pumpkin seed (la pate de citrouille;) 90 grammes of fresh seed mixed with twice as much honey—a dose which in seven hours without producing any unpleasant effect, dislodged the worm. Dr. M.'s confreres of that city, among whom were MM. Brunet, Sarramea, and others, had in a great many instances used this article with the most complete success—some of whom gave 45 grammes of the skinned seed (semences depouillees) united with the same amount of sugar. One of the physicians, who had suffered extremely for two years, from an almost constant pain in the lumbar region, excessive debility, indigestion—symptoms which he regarded as due to a disease of his nervous system. He finally, having voided some flattened fragments which he thought might be portions of tænia, took by the advice of M. Sarramea, 30 grammes of pumpkin seed pounded with 10 grammes of sugar; he suffered all night from a violent fever and great agitation; by morning, twelve hours after having taken this dose, an injection simply of water brought away seven metres, or about twenty-three feet of tænia.

As suggested by M. Rollet, measures were taken to convey to the Council of the Administration, a recommendation that this valuable remedy should be admitted into the pharmacopœia (*codex.*) (See Jour. des Connais. Med. Chir., June 1, 1852.)

*Cure for the Tapeworm.*—Procure sufficient seed of the pumpkin (those grown in the West Indies are the best) to make two ounces after removing the outside shell of the seed ; put them into a mortar and add half a pint of water ; pound them well up, and make a liquid orgeat of them, which strain through a cloth.—Drink this mixture in the morning on a fasting stomach. If it does not operate in the course of an hour and a half, take one ounce of castor oil. Drink all the time as much fresh cool water as the stomach can bear or contain ; that is, drench yourself with water. After taking the orgeat, if the stomach is well rubbed with ether, and an injection of about sixty drops of it is taken, you will find it an assistant to the orgeat, but this may not be necessary. Should the first application of the remedy not answer, repeat it the next morning, and there is no doubt your complaint will be removed. The worm will leave the patient all at once, and probably entire. This can be ascertained by finding the small end or head of it, which tapers almost to a point.

The New York friend, from whom I received the recipe, of which the preceding is a copy, in March, 1848, remarks, in support of the efficacy of this remedy, that Capt. —— says he did not have to take the injection, although he took two separate doses of the seed, (the first not operating sufficiently,) which relieved him at once, and since which time has cured probably a dozen different persons afflicted with the tapeworm, who had been given over by the physicians. The worm from him was about thirty-four feet long, and each link about one inch. He rubbed the stomach with ether after taking the orgeat. It may be advisable to use the forenamed remedy under the advice and with the assistance of a physician. I have only to add that the suffering lady in this city, for whose relief the writer's aid and influence was solicited by her husband, has been restored to perfect health, after years of prostration and efforts for relief ; and in thankfulness for the interest I had manifested in the case, sent me a glass jar containing a large part, if not the whole of the worm that had been her tormentor for several years.—*Boston Med. and Sur. Journal.*

F. W. Cragin, M. D., says : " On reading an article on pumpkin seeds, in a late number of the Boston Medical and Surgical Journal, I recommended it to an intimate friend, who had, two months before, discharged about four yards of that detested parasite, a tapeworm, and who was sure there was " more of the same sort left." He in three days afterwards, showed me the bottle, since left at your office, containing what was formerly discharged, together with the tapering part of that which was removed (in all about four yards) by the remedy.

His statements, which may be implicitly relied on, are, that for want of West Indian or other pumpkin seeds, he took undried acorn or marrow squash seeds, and proceeded, *secundum artem*, following the orgeat, in about one hour and a half, with about six drachms of castor oil, taken in two spoonsful of Holland gin. He drank very little water twice; drank and ate nothing else till noon, when the only effect of his faith and practice was manifested "in one liquid discharge containing the squirming worm; at one end about one-third of an inch broad, and tapering down to nothing."

If this remedy should continue to prove as efficacious as in this and former instances, it is to be hoped a specific has been found for one or more of "the ills that flesh is heir to;" which remedy should never be lost sight of."—*Ib.*

*On the Treatment of Tapeworm by the Oil of Pumpkin Seeds:* By the late Prof. HENRY S. PATTERSON, M. D.—In the Medical Examiner, for October, 1852, I reported a case of radical cure of tænia, by the use of an emulsion of pumpkin seeds, after the Ol. Terebinth, and even Kousso, had signally failed. Several other cases have been reported before and since mine, all going to establish the efficacy of this new remedy. Should it prove as generally successful in expelling the worm as the cases indicate, it will become a valuable accession to our means of treatment in a troublesome and often obstinate affection.

The seeds of the common pumpkin (*cucurbita pepo,*) consist of a leathery white envelop, inclosing an oily albumen of a slightly greenish tinge. They are inodorous, and have a sweetish, mucilaginous taste. Rubbed up with warm water or milk, and sweetened, they form a very pleasant emulsion; and this is the way in which they have generally been administered. They abound in fixed oil, which is readily yielded on expression, and appears to be the only constituent of any importance. Conceiving this oil to be the anthelmintic principle, I determined to use it in the first case of tænia I should encounter. A quantity was obtained, by cold expression, by our accomplished pharmaceutist, Mr. Frederick L. John, of Race street. From four lbs. of the seeds he procured ℥ xiv of oil; but, he has no doubt that, if the operation were conducted on a larger scale and more carefully, the yield would be from thirty to forty per cent. The oil is clear, transparent, of a light brownish green color, with a slight oily odor, and a perfectly bland taste, like that of the oil of sweet almonds. It has now been kept some ten months, in well stoppered bottles, and is perfectly sweet and bland.

No case of tænia has occurred in my own practice; but in May last I learned from Mr. John C. Lyons, an intelligent member of the medical class of Pennsylvania College, that a poor woman in his neighborhood (Kensington) labored under the disease, and had asked his advice. I requested him to use the pumpkin seed oil, which he did, with the happiest results. Causing her to fast rigidly for twenty-four hours, he gave her ℥ ss of the oil in the morning, and in about two hours ℥ ss more. This produced a light disposition to looseness of the bowels. In two hours after the last dose, ℥ i of castor oil was given, and purged freely, bringing away a considerable portion of the worm. From that period until the present, (September) she has remained entirely free from any symptom of verminous irritation, and there can be no doubt that the worm was altogether destroyed.

Tænia is of so rare occurrence with us, that no individual practitioner sees enough of it to enable him alone fairly to test any medicine. I therefore beg leave to call the attention of my medical brethren to a remedy readily obtained, cheap and pleasant, and which I believe will be found quite efficient.

The same gentleman reported the following case:

*Failure of Kousso—Successful Use of Pumpkin Seeds.*—The subject of this case was for some time under my care, in consultation with my colleague, Dr. Darrach. I can aver that he was most thoroughly put through the entire routine of tapeworm remedies before he left Philadelphia. He tells his own story so well that I prefer to give the following extract from a letter announcing his restoration to health: "In the early part of January, 1836, I was rather suddenly attacked with what seemed to be an alarming diarrhœa, which continued for some weeks, resisting the usual remedies. My symptoms had been peculiar for some time previous to the attack. Indeed, I had all the prominent symptoms of tænia as laid down in the books, viz: dizziness; occasional false vision; variable appetite; pain in the lumbar region; pain in the knee joint; swelling of the abdomen; hesitancy of speech; restlessness in time of sleep; unusual drowsiness during the day; variable strength, being sometimes quite strong, and then again quite feeble. Somewhere about the middle of February of the same year, I discharged at a morning stool, about nine yards of the tænia. From that time onward, for six years, I was more or less under medical treatment continuously. I took large quantities of the spts. turpentine, (once or twice two ounces at a dose;) also, the malefern, calomel, and jalap, and Jayne's vermifuge; and was several times under homœopathic treatment. I took also iodide of potassium, iodide of iron, decoction of pomegranate, and the 'kousso.' I discharged large quantities of the worm, but no head could be perceived. When the kousso failed, I began to dispair of being cured at all, but my sister, Mrs. ——, sent me in December last two numbers of the Boston Medical and Surgical Journal, containing two several accounts of the cure of tænia by the use of pumpkin seeds. Having previously abstained from usual food for a day, on the 10th of January last, I took, at 8 o'clock in the morning, two ounces of the kernels of pumpkin seeds pulverized with two tablespoonfuls of white sugar, and commingled with a half pint of boiling water, making a very pleasant drink for a fasting man. I kept my bed, drinking frequently of cold water, and at 9 1-2 o'clock, I took an ounce of castor oil.—At 10 1-2, I drank a cup of hot black tea, and, in about two minutes discharged about eight yards of the tapeworm, *with the head.* O, how I wept for joy that I was again a free man, after a servitude of six sad years to this awful complaint. Since then I feel like a new being in a new world. My life had often been a weary burden, and yet I grew fleshy and looked healthful. For months in succession I had discharged the worm daily in pieces of six to eighteen inches, and also in gourd seed form. I suppose that without any over-estimate, I discharged, during the six years of my affliction, about *four hundred yards!* The remedy is very simple. Were I a practising physician, I would never administer turpentine for tapeworm; I sometimes fear that I have experienced irretrievable harm to my kidneys by using it. There is virtue in pumpkin seeds, doctor, even if it be a *Yankee notion.*"

In the Northwestern Medical Journal, for May, 1853, Dr. J. McCreary Sudduth, gives the following case:

B. H., male, aged 28 years—occupation farmer, volunteered in 1847 to go to Mexico. In good health when he left home (Ky). Sickened while on the gulf, Nov. '47. Landed and went as far as the City of Mexico. Discharged and returned to Kentucky early in the summer of 1848, quite weak and much emaciated. In August of 1848 began to pass by stool small portions of what proved afterwards to be joints of tapeworm. Shortly after his return to Kentucky, he applied to a number of physicians for advice; receiving no benefit from any article recommended, except those used by one doctor whose prescription brought away eight feet of tapeworm. Patient thinks the articles used by him were cal. and Dr. Jayne's vermifuge. Shortly after passing this piece of worm, finding himself not relieved, and knowing now what it was that troubled him, he went to Louisville and consulted a number of physicians of that place, offering one hundred dollars to any one who would rid him of his troublesome companion. He however received no benefit from any articles used by physicians of that place. Soon after leaving Louisville, he came to Illinois, still poor and in bad health. He consulted with all the doctors that came in his way, using all articles by them recommended, as well as all the patent medicines that he saw extolled for the removal of tapeworm, (and their name is legion) in newspapers, almanacs and receipt books, &c.; however all failed. When he consulted Dr. Smick and myself, he presented the appearance and symptoms as follows:—Was lean in flesh, and ænemic, judging from the appearance of *the surface* and color of his lips. Abdomen somewhat distended, troubled much and especially at night by a moving, creeping sensation in the abdomen. Variable appetite, at times voracious, at others none at all, passing daily by stool a number of joints of the worm. We advised him to eat the paste of pumpkin seed and honey, ℥ iij, one ounce at a time, with an interval of two hours. Six hours after taking the last portion he passed twenty-two feet of the worm, though in three pieces, the longest of which was eighteen feet, and bearing the head of the parasite; showing the superiority of paste of pumpkin seed and honey over all known articles for the removal of this troublesome, if not destructive parasite. All symptoms of worms have disappeared.

In the American Journal of the Medical Sciences for July, 1854, D. Leasure, M. D., of Newcastle, Pa., says: "Mary ——, aged twenty-eight, unmarried, has been delicate all her life, and for fifteen years subject to severe cramping pains of the abdomen, accompanied sometimes, with obstinate vomiting. About ten years since, she noticed that she passed portions of tapeworm, of lengths varying from a single joint up to many feet, and if the statements of the patient and her mother are to be relied on, sometimes half filling an ordinary chamber mug. Her mother had also, at an early period of her life, been a victim to a tapeworm, which had been expelled by a secret *vegetable remedy*, probably *male fern*, given to her by a worm doctor.

My attention was called to Mary's case some time in last February, while in attendance on her sister, for another disease; but from causes unnecessary to mention, I did not prescribe till last week. I had intended to use the male fern or kousso, or both; but not having access to either of them in a fresh state, I determined to wait till they could be procured from Philadelphia.—While thus waiting, I noticed in one of the journals a report of a case of tænia expelled by the use of emulsion of pumpkin seeds. Curiosity, more than the expectation of success, prompted me to give it a trial. I directed a pint of the bruised seeds to be infused in three pints of soft boiling water, and left over night, the whole to be taken during the next day, the patient fasting in the meantime.

On the morning of the 9th of May, the patient commenced its use, and in the afternoon experienced the most violent cramps and pains in the bowels for several hours; and on the morning of the 10th, she passed eleven feet of the parasite, including the head, as proved by observation under the microscope. The animal was entirely dead when voided from the bowel, and is a most beautiful specimen of a perfect tænia—*N. Orleans Med. & Sur. Journal.*

